# Extended Signal-Space Separation Method for Improved Interference Suppression in MEG

**DOI:** 10.1109/TBME.2020.3040373

**Published:** 2021-06-17

**Authors:** Liisa Helle, Jukka Nenonen, Eric Larson, Juha Simola, Lauri Parkkonen, Samu Taulu

**Affiliations:** Department of Neuroscience and Biomedical Engineering, School of Science, Aalto University, Espoo, Finland, and also with MEGIN Oy, 00076 Helsinki, Finland; MEGIN Oy.; Institute for Learning and Brain Sciences, University of Washington.; MEGIN Oy.; Department of Neuroscience and Biomedical Engineering, School of Science, Aalto University, and also with MEGIN Oy.; Institute for Learning and Brain Sciences, University of Washington, and also with the Department of Physics, University of Washington.

**Keywords:** External interference, interference suppression, magnetoencephalography, principal component analysis, signal processing, signal-space separation

## Abstract

**Objective:**

Magnetoencephalography (MEG) signals typically reflect a mixture of neuromagnetic fields, subject-related artifacts, external interference and sensor noise. Even inside a magnetically shielded room, external interference can be significantly stronger than brain signals. Methods such as signal-space projection (SSP) and signal-space separation (SSS) have been developed to suppress this residual interference, but their performance might not be sufficient in cases of strong interference or when the sources of interference change over time.

**Methods:**

Here we suggest a new method, extended signal-space separation (eSSS), which combines a physical model of the magnetic fields (as in SSS) with a statistical description of the interference (as in SSP). We demonstrate the performance of this method via simulations and experimental MEG data.

**Results:**

The eSSS method clearly outperforms SSS and SSP in interference suppression regardless of the extent of *a priori* information available on the interference sources. We also show that the method does not cause location or amplitude bias in dipole modeling.

**Conclusion:**

Our eSSS method provides better data quality than SSP or SSS and can be readily combined with other SSS-based methods, such as spatiotemporal SSS or head movement compensation. Thus, eSSS extends and complements the interference suppression techniques currently available for MEG.

**Significance:**

Due to its ability to suppress external interference to the level of sensor noise, eSSS can facilitate single-trial data analysis, exemplified in automated analysis of epileptic data. Such an enhanced suppression is especially important in environments with large interference fields.

## I. Introduction

**M**AGNETOENCEPHALOGRAPHY (MEG) is a neuroimaging method that aims to reconstruct neural current distributions in the brain, based on a multi-channel measurement of the magnetic field distribution outside the head. While MEG can, under favorable conditions, reach sub-centimeter spatial resolution, the source reconstruction accuracy is often compromised by the interfering magnetic fields that may be significantly stronger than the weak magnetic fields produced by the brain. Consequently, efficient suppression of interference is essential to ensure reliable analysis of neuromagnetic fields.

Currently, several efficient computational methods exist for the suppression of such interfering fields. These methods may utilize signals from reference sensors measuring the external interference outside of the main sensor array [1] together with e.g. modelling based on independent component analysis (ICA) [2], [3]. Interference can also be suppressed without dedicated reference sensors; such methods can operate: i) in the spatial domain, such as signal-space projection (SSP) [4], signal-space separation (SSS) [5], [6] and generalized sidelobe canceller [7], ii) in the temporal domain, such as temporal filtering, iii) in the spatio-temporal domain, such as spatiotemporal SSS (tSSS) [8], [9] and dual signal-subspace projection (DSSP) [10], or iv) in the frequency domain, such as spectral signal-space projection (S3P) [11]. In this work, we will concentrate on the interference suppression in the spatial domain.

The SSS and SSP methods suppress spatial magnetic field patterns that have *a priori* been classified as interference. Whereas the SSS method relies on the physics-derived differences between the field patterns of interest (i.e. sources inside the sensor helmet) and those of interfering sources (i.e. sources outside of the sensor helmet), the SSP method is based on a statistical analysis of on-site interference measurements. Although the SSS method is efficient and generally applicable against any external interference fields generated by sources farther than approximately 0.5 m from the sensors, its shielding factor (SF, see II-G3) is limited by inaccuracy of the sensor calibration and geometry information. On the other hand, the shielding factor of SSP can become very high, but only against known, measured external interference patterns.

In this paper, we propose a new interference suppression method, termed extended SSS (eSSS), that efficiently combines the main ideas of the SSS and SSP methods. It is designed to provide i) the generality of SSS towards all interference field patterns, and ii) insensitivity to small sensor calibration and position inaccuracies due to the statistical information similar to that used in SSP. Here, we show that the eSSS method can provide superior interference suppression compared to the aforementioned commonly-used methods without introducing bias to the brain signals.

## II. Methods

### A. Interference Suppression in the Signal Space

In spatial modeling of the multichannel MEG data, the response of the MEG system to a magnetic field distribution at any given moment is often represented as an *N*-dimensional signal vector

(1) $$\phi ={\left[\phi_{1}\;\ldots \;\phi_{N}\right]}^{\text{T}},$$

where *N* is the number of sensors in the MEG system, *ϕ*_*j*_ corresponds to the output of the *j*:th sensor, and (·)^T^ stands for transpose. The sensor-level signals can be represented in terms of the lead fields which describe the coupling of the underlying neural current distribution to the MEG sensors [12]. Lead fields generally overlap between sensors; therefore, the number of free parameters in the spatial model, which describes a measured signal vector due to a current distribution, must be smaller than *N*. Modern multichannel MEG systems provide generous oversampling of the detectable magnetic field [13], affording the ability to construct a principled spatial model for MEG with fewer degrees of freedom than *N*.

### B. Physics-Based Model: Signal-Space Separation

A commonly-used spatial model for representing the multichannel MEG data is the signal-space separation (SSS) method [5], [6]. SSS is based on i) the validity of the quasi-static approximation of Maxwell’s equations for the magnetic fields detected by MEG systems and ii) the location of MEG sensors in a source-free volume [5].

In the SSS framework, the signal vector ***ϕ*** can be represented as a unique linear combination of basis vectors derived from a set of basis functions called vector spherical harmonics (VSH). In addition, the basis functions of signals arising from the brain (the space inside the sensor array in the MEG measurement geometry, referred to as *in*) and from the external interference sources (arising from the space outside of the sensor array, referred to as *out*) differ in their convergence properties and can therefore be separated into two signal subspaces [5]. Thus,

(2) $$\phi =\sum_{l=1}^{L_{\text{in}}}\sum_{m=-l}^{l}\alpha_{lm}a_{lm}+\sum_{l=1}^{L_{\text{out}}}\sum_{m=1}^{l}\beta_{lm}b_{lm},$$

where **a**_*lm*_ and **b**_*lm*_ are derived from spherical harmonic functions and form the internal **S**_in_ and external **S**_out_ SSS bases, respectively.

(3) $$S=\left[S_{\text{in}}\;S_{\text{out}}\right],$$

and the coefficients *α*_*lm*_ and *β*_*lm*_, called magnetostatic multipole moments, are collected in **x**. Thus,

(4) ϕ=Sx

Note that the *l* = 0 term has been omitted as it vanishes for the magnetic fields. The multipole moments are independent of the sensor geometry and can be estimated as

(5) $$\hat{x}=\left[\begin{array}{c} {\hat{x}}_{\text{in}}\\ {\hat{x}}_{\text{out}} \end{array}\right]=S^{†}\phi ,$$

where the pseudoinverse **S**^†^ = (**S**^T^**S**)^−1^**S**^T^. The reconstruction of the biomagnetic signals arising inside of the sensor helmet is thus

(6) $${\hat{\phi }}_{\text{in}}=S_{\text{in}}\;{\hat{x}}_{\text{in}},$$

where the suppression of external interference magnetic fields is accomplished by neglecting the latter expansion in (2). Because the dimension of the SSS basis *< N* and the SSS basis is linearly independent for practical MEG arrays, the decomposition between magnetic fields originating from inside and outside of the sensor helmet is unique even though the corresponding basis vectors are not mutually orthogonal [5]. Due to the fast distance-decay of high spatial frequencies, which correspond to high expansion orders, the expansion orders *L*_in_ = 8 and *L*_out_ = 3 in (2) are, in practice, sufficient for MEG data recorded with SQUID-based sensors that are located at a distance of more than 3 cm from the cortex [5].

#### 1) Generality:

Based on the physics of magnetic fields, the SSS method is both general and versatile and does not assume that sources of external interference magnetic fields remain constant. However, the application of the SSS model requires truncation of the VSH expansion in (2), leading to modest suppression against sources that are closer than approximately 0.5 m to the nearest MEG sensor [5].

#### 2) Sensitivity to Errors in Calibration and Measurement-Geometry:

The SSS method depends on establishing an accurate model of external magnetic fields. It is therefore sensitive to the knowledge of the sensor geometry, such as the locations and orientations of the sensors, the areas of the pick-up coils, and the imbalance between the loops in gradiometers, as well as the sensor calibration coefficients [5], [14], [15]. Also cross-talk between MEG channels affects the SSS performance, if not properly corrected [5].

In the ideal case of both an infinitely accurately measured magnetic field, and high enough expansion orders (*L*_in_ and *L*_out_) in the SSS model, separation of the multipole moments into the internal **x**_in_ and external **x**_out_ contribution is perfect (see (5), (6)). Thus, SSS would not result in any residual fields and the shielding factor, describing the method’s ability to suppress external interference, would be very high. However, if the uncertainty of the sensor calibration is e.g. 1%, the ensuing mixing of **x**_in_ and **x**_out_ reduces SF to ∼ 15 [5]. If the calibration accuracy is instead improved to 0.1%, which can be reached in practice with the so-called fine-calibration correction, SF increases to 150–200 [5], [16]. However, in electromagnetically harsh environments, such as in a light-weighted magnetically shielded room (MSR) in an urban setting, this level of interference suppression is not sufficient.

### C. Statistics-Based Model: Signal-Space Projection

Another widely-used method for interference suppression in multichannel MEG is signal-space projection (SSP) [4], [17]. In this framework, the dominant interference signal-space directions are defined by using principal component analysis (PCA), which is typically computed from a recording conducted without a study subject in the MSR (empty-MSR recording). Data measured with a subject are then projected onto a basis that is orthogonal to the noise subspace spanned by the interference components,

(7) $$P_{⊥}\phi =P_{⊥}\left(\phi_{\text{in}}+\phi_{\text{out}}\right)=P_{⊥}\phi_{\text{in}}+0=P_{⊥}\phi_{\text{in}},$$

where we have assumed that ***ϕ***_out_ is spanned by the PCA-based interference subspace, which we will call **U**. The orthogonal SSP operator **P**_⊥_ is constructed as

(8) $$P_{⊥}=I-UU^{T}$$

where **I** is the identity matrix.

However, the signal subspaces spanned by the possible source distributions inside and outside of the sensor helmet are not orthogonal. As a result, the spatial projection method can distort the brain signals and this distortion must be compensated for in source modelling [4], [18].

The SSP method may also require selection of the number of principal components (PCs) for the interference suppression. With a helmet-like sensor array, 8–12 dominant components are usually enough to span the subspace that contains the interference. This relatively small number is consistent with the fact that typically the amplitude of environmental interference decreases with increasing spatial complexity. Therefore, most of the signal power will be in the homogeneous and first-order gradient components that occupy three and five degrees of freedom, respectively.

#### 1) Limitations Due to Instability of Interference Sources:

As the spatial model that is applied in the SSP method is determined on the basis of a recording performed without a subject, the main limitation of the SSP method is the assumption of interference conditions remaining similar during that separate empty-MSR recording and the actual measurements. Any subsequent changes in the interference patterns will result in a compromised shielding factor. For SSP to be effective against new interference sources, it should be recomputed based on a new recording. The sources of strong external interference fields, however, tend to remain stationary, and the MSR aligns residuals of external magnetic fields along its axes regardless of their pattern outside the room, which partly alleviates this problem [16]. In contrast, the MSR does not homogenize the shape of interference fields produced by sources inside the MSR.

#### 2) Insensitivity to Calibration and Sensor-Geometry Errors:

Since the SSP method is based on a statistical rather than physical model of the data, the method is insensitive to sensor calibration, position and orientation, gradiometer imbalance, as well as to cross-talk between the channels, provided that these parameters remain constant. For stable interference patterns, SSP can achieve higher shielding factors than SSS. However, for efficient interference suppression, SSP requires that the amplitude response of the sensors has to be highly linear [4], i.e., the magnetic field pattern due to a spatially stable source should remain the same irrespective of the amplitude of that source. This is typically true for SQUID sensors that employ negative feedback and thus provide sufficient linearity, however, this might not be the case for other sensor types.

### D. Novel Extended Signal-Space Separation, eSSS

We propose an extension of the SSS method that adds statistical aspects of the data to the SSS model in order to improve the accuracy of the interference basis [19]. The extended SSS (eSSS) method retains the generality of SSS but reduces its sensitivity to geometry- and calibration errors. This is accomplished by augmenting the external basis with the portion of the statistically determined interference that is not spanned by the computational SSS basis. Our new method is thus expected to increase the shielding factor of SSS and SSP against all external interference.

The dominant principal components (**PC**_out_) are determined from an unprocessed empty-MSR recording according to the SSP framework and added to the external SSS basis **S**_out_ to form an extended external SSS basis as

(9) $$S_{\text{out,e}}=\text{orth}\;(\left[S_{\text{out}}\;{\mathrm{PC}}_{\text{out}}\right]).$$

The external SSS basis **S**_out_ and the principal components **PC**_out_ are generally not orthogonal to each other but are orthogonalized here (denoted above as orth(·)), for example, by the singular value decomposition (SVD), in order to prevent singular **S**_out,e_. This step is necessary because some of the computationally and experimentally determined interference patterns can be arbitrarily close to each other in terms of signal-space directions. After orthogonalization, the extended external SSS basis **S**_out,e_ includes, in addition to the **S**_out_, features that correct the inaccuracies due to calibration and geometry errors. The whole extended SSS basis **S**_e_ can be then constructed as in (3) but using **S**_out,e_, as

(10) $$S_{\text{e}}=\left[S_{\text{in}}\;S_{\text{out,e}}\right],$$

and used as in the conventional SSS processing, i.e., the data are first decomposed to the coordinates $\hat{x}$ (see (5)) in the predetermined eSSS basis **S**_e_ (10), and reconstructed back to sensor signals as in (6) excluding the external fields. However, this reconstruction of the signals arising from sources inside of the sensor helmet will now provide cleaner brain signals due to the better description of external magnetic fields. Due to the fact that most of the signal power of the magnetic field will be in the homogeneous and first-order gradient components occupying three and five degrees of freedom, respectively, we have used here 8 dominant PCs in eSSS model.

In addition, the SSS basis can be extended by artifact features not belonging to the ideal computational SSS basis. For example, PCs can be estimated from unprocessed data band-pass filtered to the frequency band of an artifact not suppressed by eSSS, yielding **PC**_out,f_; using

(11) $$S_{\text{out},\text{e}}^{\prime }=\text{orth}\left(\left[S_{\text{out}}\;PC_{\text{out}}\;PC_{\text{out,f}}\right]\right)$$

results in eSSS′-processed data. Depending on the spatial complexity of the artifact, fewer vectors might be required for **PC**_out,f_ than for **PC**_out_ to represent the artifact sufficiently.

Similarly to SSS, the eSSS method applies an oblique projection to separate the linearly independent internal and external spaces. As a result, eSSS does not project out nor bias brain signals. However, as eSSS adds interference features to the SSS basis, it can increase the condition number of the basis (the ratio between largest and smallest matrix singular value). This, in turn, can result in increased reconstruction noise in the processed data. Therefore, adding unnecessary or otherwise too many vectors to the basis should be avoided.

### E. Simulations

We performed simulations to test the efficacy of the eSSS method. The magnetic field was simulated using the SSS basis and the simulation data set included an empty-MSR simulation of external interference (Simulation 1; Table I) and source simulation series (Simulations 2 and 3; Table I). The source simulations comprised a dipolar source within the sensor helmet with (Simulation 2) and without (Simulation 3) the presence of external interference. In the former case, the interference was either “known” (Simulations 2a), i.e., similar to that in Simulation 1, or “unknown” (Simulations 2b), i.e., containing patterns not present in Simulation 1.

All simulations contained sensor noise with spatially uncorrelated and correlated parts, with the total noise approximately at the level of typical sensor noise for an MEG device. Simulation 1 was 1-min long and all simulations were sampled at 1 kHz. We used the fine-calibration information obtained from an existing MEG device in the generation of all the simulations to mimic a real situation where sensor geometry and calibrations are not perfectly known. We performed the analysis using 0.5% error in this calibration as well as without using the fine-calibration information at all.

For Simulations 2 and 3, current dipoles were individually activated with a peak amplitude of 1000 nAm by one cycle of a 10-Hz sinusoidal signal followed by 100 ms of zero signal. Altogether 100 such 200-ms long trials were simulated, totaling of 20 s of data per dipole. A total of 70 dipoles were simulated at random locations in the upper hemisphere (*z >* 0 in the MEG device coordinates), with depths varying from 10 to 70 mm from the origin, in steps of 10 mm. Thus, we had 10 dipoles at each depth, all at tangential plane of the sphere. These simulated source signals were identical and only the presence and type of the external interference were varied.

The external interference in Simulations 1–2 contained homogeneous magnetic fields (*L*_out_ = 1 in the SSS-model) at a low frequency (*<*2 Hz), and first-order gradient magnetic fields (*L*_out_ = 2 in the SSS-model) at a higher frequency (∼ 20 Hz). The exact frequencies differed between Simulations 1 and 2. The amplitude of the total external interference was roughly the dynamic range of the magnetometer signals (± 20 nT) of the TRIUX™ neo MEG system (MEGIN Oy, Helsinki, Finland). The amplitude of the first-order gradient magnetic field was approximately 10% of that of the homogeneous field.

Simulation 2a had the same, non-zero amplitudes for the external multipole moments as Simulation 1, yielding the same direction of the total external magnetic field. On the contrary, Simulation 2b contained homogeneous magnetic field components that were not present in Simulation 1, mimicking the case of a new interference source appearing, i.e., signals that are not present in the empty-MSR data.

### F. Measurements

#### 1) Phantom:

To verify the detectability of brain sources and unbiased source estimates after eSSS processing, we recorded signals from a dry phantom (MEGIN Oy, Helsinki, Finland) that was positioned in the MEG sensor helmet. This phantom contains triangular current loops with two long, radially-oriented sides and tangential base. This construction generates a field equivalent to that of a tangential primary current residing inside a spherically symmetric volume conductor while the radial segments of the triangles represent the contribution of associated volume currents.

A total of 32 current dipoles in the phantom were excited with 2 cycles of a 20-Hz sinusoidal signal, and 100 trials from each dipole were collected. The peak dipole amplitude was 500 nAm, the dipoles were located 34 mm (deep source) to 64 mm (superficial source) from the sphere origin. Naturally, the data also contained ambient external interference. These recordings (Measurement 1; Table II) were performed with a TRIUX™ system (MEGIN Oy, Helsinki, Finland) at the MEG manufacturing facility; an urban environment with strong external interference. In addition to the phantom data, we recorded 3 minutes of empty-MSR data for the determination of PCs to be used in the SSP and eSSS processing.

#### 2) External Interference:

Three minutes of empty-MSR data with very strong, naturally-occurring external interference, mainly due to car traffic on nearby streets, were measured at factory (Measurement 2; Table II) and used to estimate the obtained shielding factors. Additional empty-MSR data for the estimation of the PCs were also measured in the same session, resulting in a situation comparable to the “known” interference in Simulation 2a.

#### 3) New External Interference:

In order to test the performance of eSSS in the case of new interference sources, we also measured data where a large magnetic object (manual forklift) was moving outside of the MSR (Measurement 3; Table II). This introduced a new type of an artifact during the measurement (cf. “unknown” interference in Simulation 2b), not present in the additional empty-MSR data that were also collected during this measurement session for the estimation of PCs.

#### 4) Measurement With Internal Active Shielding:

The measurements above were performed in a MSR with passive shielding only. However, several MEG sites require additional active compensation techniques, such as external and internal active shielding (IAS) to keep all sensors within their dynamic range [16]. Therefore, we also recorded phantom and empty-MSR data with IAS active (Measurement 4; Table II).

#### 5) Vibration of the MSR:

Mechanical vibration of the MSR floor and walls in an external magnetic field can induce artifacts within a specific frequency band (usually between 10–30 Hz) [16]. If the whole MEG sensor helmet moves in synchrony due to the vibration, the generated field pattern is that of an external field, which is efficiently suppressed by SSS and causes no ensuing problems in the data analysis. If, for some reason, some of the sensors or any magnetic components in the system move in an asynchronous fashion, SSS is unable to suppress the artifact because its spatial pattern is not spanned by the computational external SSS basis. To study the effectiveness of eSSS against vibration-related artifacts, an empty-MSR measurement was performed at a site where a chiller mounted to the ceiling just below the MSR caused artifact bursts in MEG recordings (Measurement 5; Table II).

#### 6) Auditory Evoked Fields:

We used auditory evoked fields (AEF) with strong external interference to demonstrate the effect of eSSS in human brain signals (Measurement 6). Data have been reported earlier [9], and subject, stimuli, data collection, and creation of additional external interference are described in detail in [9] (ii. External interference condition). The study had a prior approval by the Ethics Committee of the Hospital District of Helsinki and Uusimaa and the experiment was conducted in accordance with the Declaration of Helsinki. The empty-MSR data used in eSSS processing had been collected in the same measurement session but without the artificially-generated external interference, corresponding to the situation in Measurement 3.

### G. Data Processing

#### 1) Interference Suppression:

In SSS processing, the default expansion orders (*L*_in_ = 8, *L*_out_ = 3) were used and the expansion origin was in the middle of the sensor helmet. Cross-talk between channels occurring in the real data was corrected using the measured cross-talk correction matrix, and site-specific fine-calibration information was applied in the SSS processing. In the case of simulated data, the fine-calibration information had 0.5% error compared to the calibration used in the simulation. Analysis was also performed without fine-calibration information.

The PCs for SSP were defined from 2-minute segments of empty-MSR recordings (1-minute segment in the case of a simulation), and five most significant signal-space directions were determined both for gradiometers and magnetometers, separately. The SSP was always applied to different data than the data PCs were estimated from.

In eSSS processing, the same parameters were utilized as in SSS modelling. In defining the extended basis, 2 minutes of data (1 minute in simulation) were used for the determination of eight PCs, which were defined jointly for gradiometers and magnetometers, using a scaling factor of 100 for magnetometers to have a similar numerical range in both sensor types. The extended external basis in (9) was orthogonalized using SVD.

In the case of the mechanical vibration artifact, the eSSS′ basis $S_{\text{e}}^{\prime }$, as defined in (11), was augmented from the 8 empty-room PCs by 3 additional PCs derived from 20 seconds of band-pass-filtered data at the frequency of the vibration artifact (16.8–18.8 Hz).

All signal processing was performed using custom Matlab and Python scripts, the latter ones running on the MNE-Python platform [20], [21].

#### 2) Signal Processing and Source Estimation:

The fast Fourier transform (FFT) method was used to estimate the amplitude spectra (Welch’s method using a 4096-point Hann window and 50% overlap).

Source estimates were obtained by fitting current dipoles in the least-squares sense [12] using the MNE-Python software [20], [21]. In the case of SSP-processed data, SSP was applied also to the forward computation to compensate for signal distortion. With simulated and phantom data, a spherical conductor model with the origin at the known phantom/simulation “head” origin was employed. All channels were used and channels were weighted based on the full noise covariance matrix, which has been shown to reduce localization error compared to using the simplistic diagonal noise assumption [22]. The noise covariance was estimated from the unaveraged baseline periods of the data, and the dipole fitting was performed at the peak of the sinusoidal wave on trial-averaged data. The source localization error was calculated as the mean Euclidean distance between the known and the estimated dipole locations, and the amplitude error was calculated as the mean of absolute difference between real and estimated source amplitudes, relative to the known amplitude.

In case of AEFs, we first performed dipole fitting to averaged evoked responses at the peak of the N100 response, for left and right hemisphere separately, using approximately half of the sensors (174) at that side of the helmet. A spherical conductor model was employed, and full noise covariance was estimated from the unaveraged pre-stimulus periods. Using SSS-processed and low-pass-filtered (at 40 Hz) data, we then determined the peak N100 latency of each (unaveraged) trial. For this we used the global field power (GFP) of 16 gradiometers on top of the left auditory cortex showing the largest N100 deflection in the average AEF. We searched for the amplitude maximum within ± 20 ms around the peak latency of the averaged N100 response and by visual inspection accepted epochs with a distinct response peak in GFP and a bilateral dipolar fieldmap. We then fitted current dipoles to unaveraged SSS- and eSSS-processed data at the determined latencies using the same channel selection as previously with the averaged left-hemisphere AEFs. We used the average Euclidean distance from the estimated single-trial dipole fit locations to the mean of these locations across trials as the quality measure.

#### 3) Shielding Factor:

Shielding factor (SF) quantifies the level of suppression of external interference. SF was estimated during a large-amplitude swing of the data as the ratio of the norms of the measured (***ϕ***_raw_) and processed (***ϕ***_proc_) magnetometer signal vectors [14], i.e.,

(12) $$\text{SF}=\frac{\left\|\phi_{\text{raw}}\right\|}{\left\|\phi_{\text{proc}}\right\|}.$$

Here we used averaged SF values over a period of 2 s during the largest swing of the data. In the case of a simulation, we defined SF as the ratio of the FFT peak amplitudes at a specific frequency as

(13) $${\text{SF}}_{\text{sim}}=\frac{\text{FFT}\left(\phi_{\text{raw}}\right)}{\text{FFT}\left(\phi_{\text{proc}}\right)}.$$

This way the simulated internal signal at different frequency did not interfere the results. However, these values are comparable to those estimated with (12).

As SF depends on the strength of the external magnetic field, SF-comparisons between different processing methods are relevant when using the same measured or simulated data. It should also be noted that the SF value will saturate to a level at which the original interference signal reaches the dynamic range of the sensors or the residual interference level falls below the sensor noise. Therefore, our analysis on the SF performance of different methods will be limited by the amplitude of the interference and by the sensor noise.

## III. Results

### A. Simulations

#### 1) Suppression of External Interference:

Interference suppression provided by the SSP, SSS and eSSS methods are shown in Table III in the two simulated conditions of external interference (Simulation 2a and 2b). In line with earlier results [6], SF provided by the SSS method is limited by the calibration accuracy and thus the fine-calibration adjustment increases SF. The same applies to the eSSS method if the external magnetic field is completely “unknown” and therefore absent from the eSSS basis; in this case eSSS practically reduces to SSS.

When the external interference is “known”, SF of eSSS becomes considerably higher than that of SSS, reaching SF of SSP. In addition, the lack or fine-calibration information has only a minor impact on the eSSS performance.

**Fig. 1.**
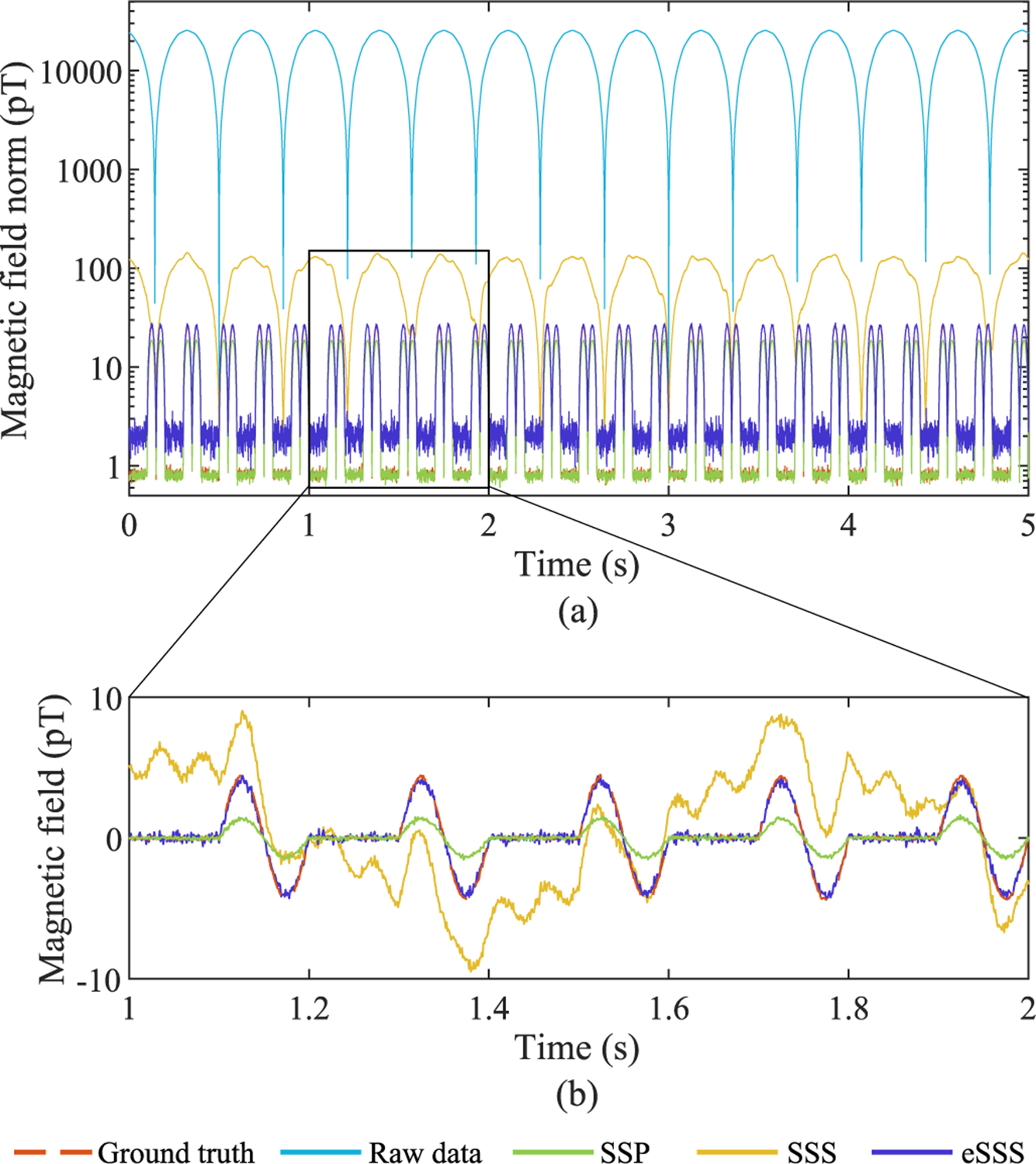
Interference suppression, Simulation 2a. The periodic low-frequency signal is the interference, and the 10-Hz single-cycle signal visible after SSP and eSSS processing is from the simulated dipolar source. (a) Norm across all magnetometers. (b) Sample magnetometer signal.

Fig. 1 shows the norm across all magnetometers and a sample magnetometer signal (Simulation 2a, dipole depth 50 mm) for comparison of different processing results with the ground-truth signal (Simulation 3). In this case, both SSP and eSSS are able to remove the interference, thus the higher SF of SSP compared to eSSS (Table III) is not due to higher residual interference fields in eSSS data, but rather to different noise properties of these processed data sets, visible in Fig. 1. It is known that SSS – and thus also eSSS – decreases the noise level in gradiometers while increasing it slightly in magnetometers, and SSP reduces noise in all sensor signals, explaining these results when magnetometers only are considered. Furthermore, suppression of the simulated source signals after SSP processing is visible in Fig. 1, whereas eSSS reproduces the simulated source signal without distortion.

#### 2) Conservation of Internal Source Signal:

**Fig. 2.**
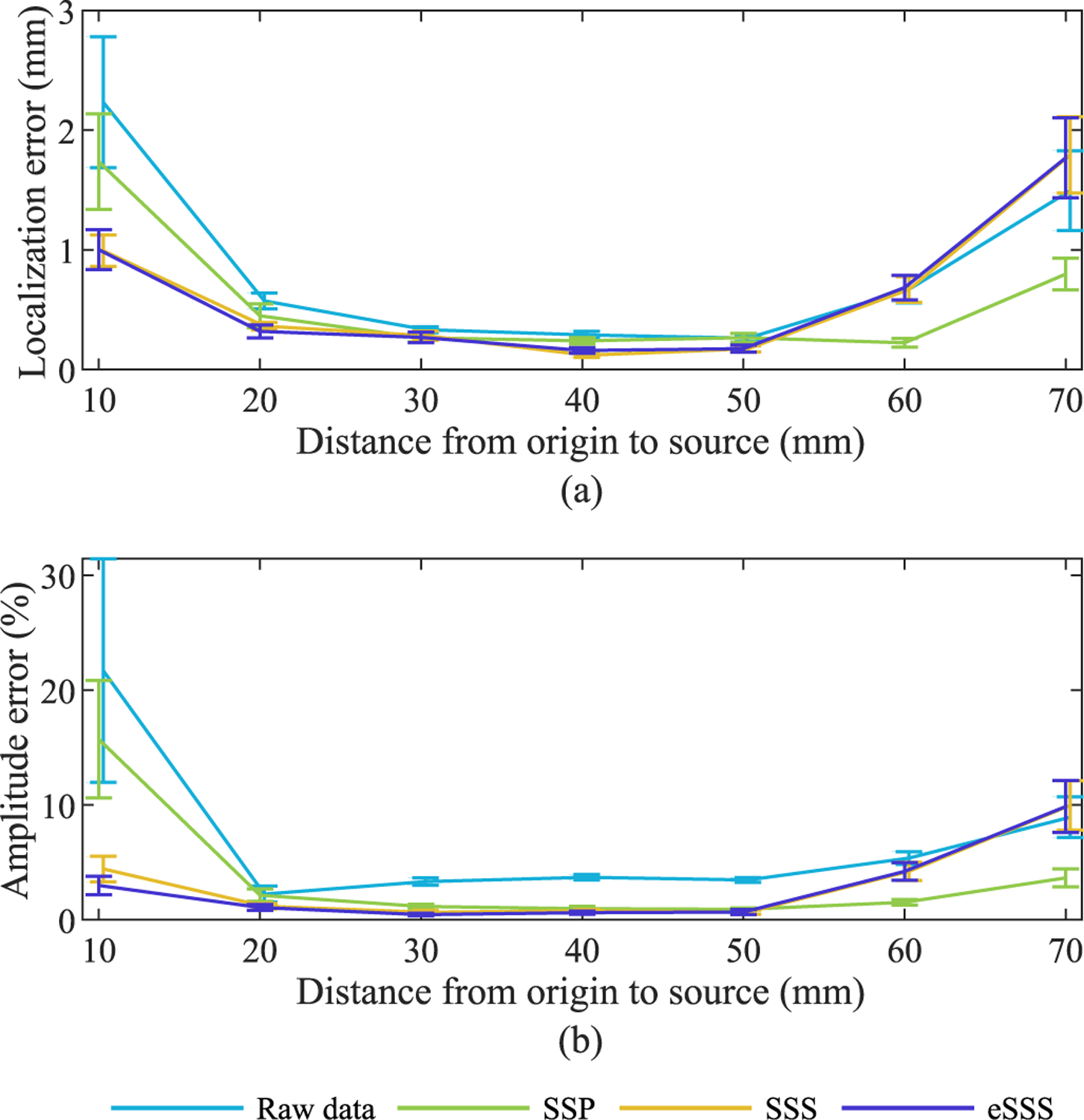
Average source estimation errors with standard error of mean. (a) Localization error. (b) Amplitude error. Simulation 2a.

Source estimation errors are shown in Fig. 2 as a function of dipole depth (Simulation 2a). The localization error remains below 2 mm for SSS and eSSS in all simulated dipole depths, exceeding 1 mm only in the most superficial dipole. On the other hand, SSS and eSSS outperform SSP on the deepest sources, where SSP produces localization error of about 2 mm and amplitude error of about 15%. Amplitude error for eSSS also coexists with localization error at the most superficial dipole (10%), but does not exceed that of SSS and remains below 5% at all other dipole depths.

### B. Measurements

#### 1) Suppression of External Interference:

**Fig. 3.**
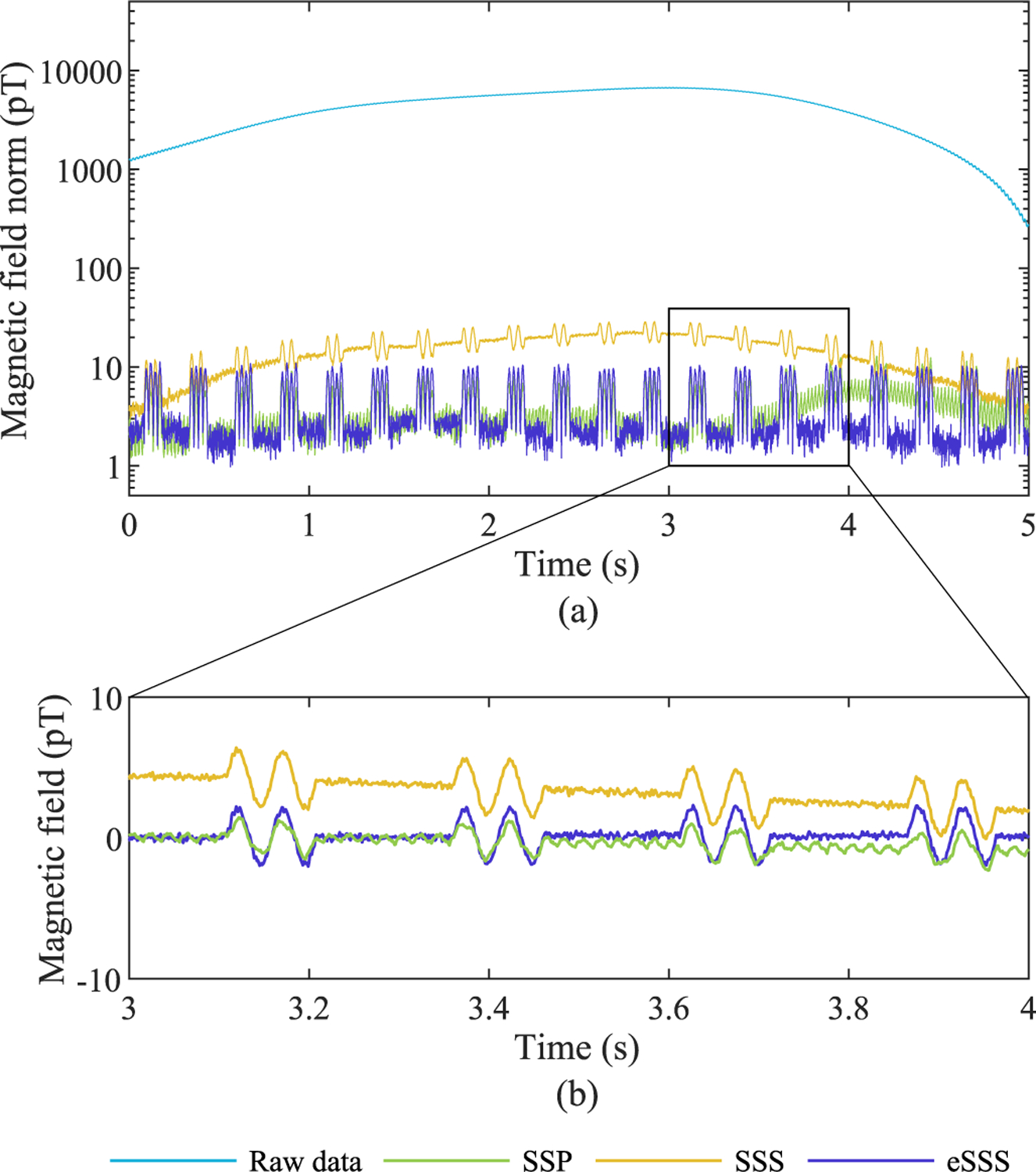
Interference suppression, Measurement 2. (a) Norm across all magnetometers. (b) Sample magnetometer signal.

Table IV lists the SFs obtained for real MEG data (Measurement 2) and shows that eSSS (with fine-calibration information) outperforms SSP and SSS. A similar result is illustrated in Fig. 3, where barely detectable phantom dipole signals (depth 34 mm) become clearly visible after the SSS, SSP, and especially eSSS processing. Whereas SSP-processed data still exhibit some residual external interference, eSSS processing reduces this interference to the level of sensor noise. Furthermore, in line with the findings in simulations (Fig. 1), SSP seems to suppress the phantom signals compared to eSSS- or SSS-processing results.

#### 2) Suppression of New External Interference:

Table V compares the SFs obtained from Measurement 3 in cases of “known” vs. “unknown” interference. Again, eSSS outperforms other processing methods, even in the case of “unknown” interference, where eSSS is able to suppress a large part of the external interference unlike SSP, where the SF remains modest. Note that the apparent difference in the SFs provided by eSSS in Table IV (with fine calibration) and Table V (interference “known”) is only due to the different interference level in the raw data, as the processing was identical and there were no residual field left in either of the eSSS-processed data.

#### 3) Suppression of External Interference in Case of IAS Active:

Measurement 4 was performed with IAS active (data not shown). Despite the significantly weaker external interference field (152 pT) due to the active shielding, a small residual interference field was visible after SSS processing (9 pT). In addition, unlike in the case of IAS off, eSSS was not able to suppress external interference to the level of sensor noise, but left a small residual field (6 pT) when IAS was active (cf. norm *<* 4 pT in Fig. 3 when IAS was off).

#### 4) Suppression of MSR Vibration Artefacts:

**Fig. 4.**
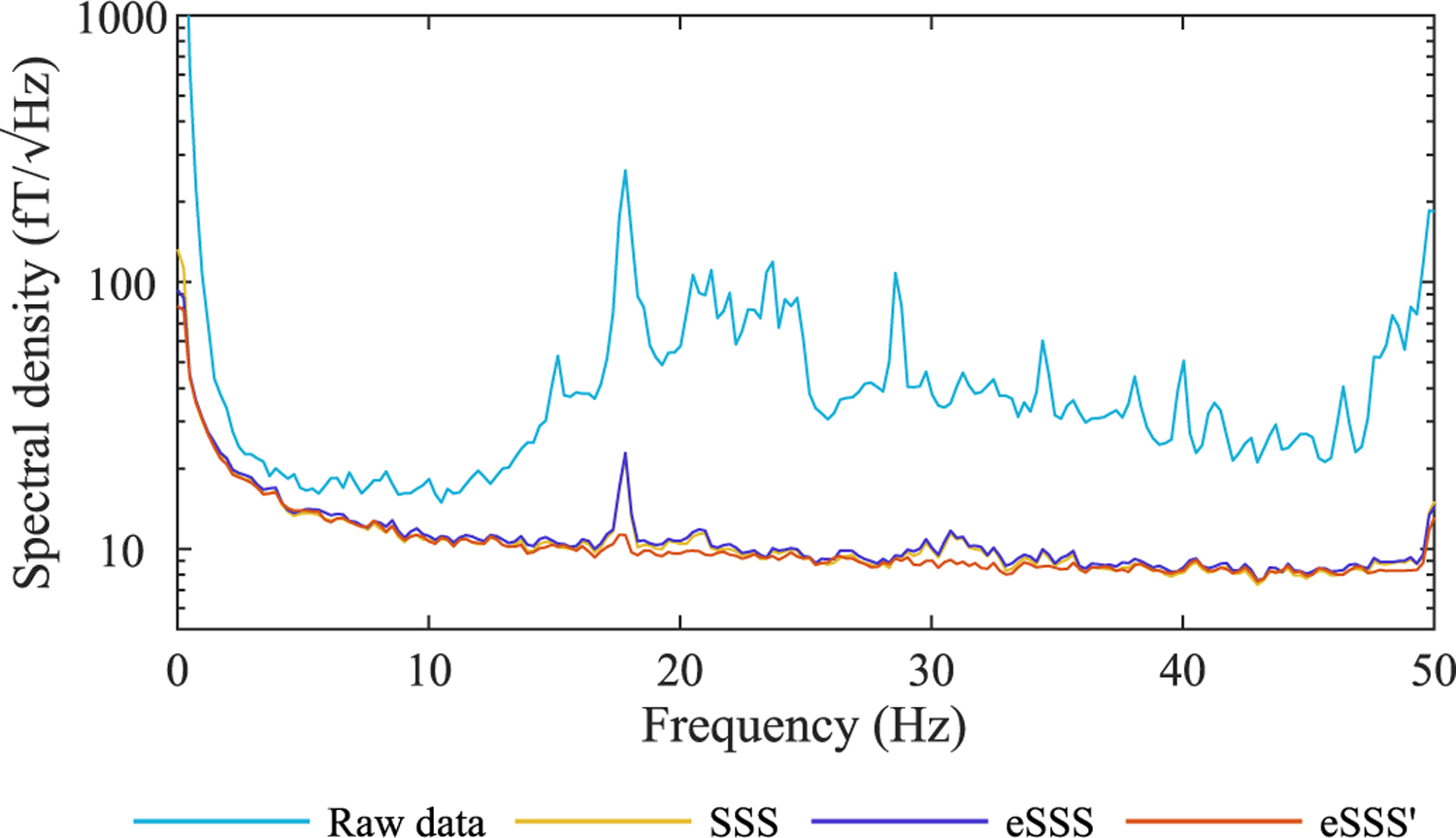
Average spectral density of magnetometers showing artifact from a MSR vibrating at 17.8 Hz. Measurement 5.

The data recorded in an MSR during a burst of mechanical vibrations (Measurement 5) had interference signals in a wide frequency band where the 17.8-Hz component was the dominant one (Fig. 4). This artifact peak was still present in the SSS-, as well as eSSS-processed data, suggesting that further extension of the interference basis could be effective. Fig. 4 displays the resulting spectra where eSSS′ tuned for this artefact has suppressed it almost completely, performing clearly better than any other spatial interference suppression method shown here.

#### 5) Conservation of Internal Source Signal:

**Fig. 5.**
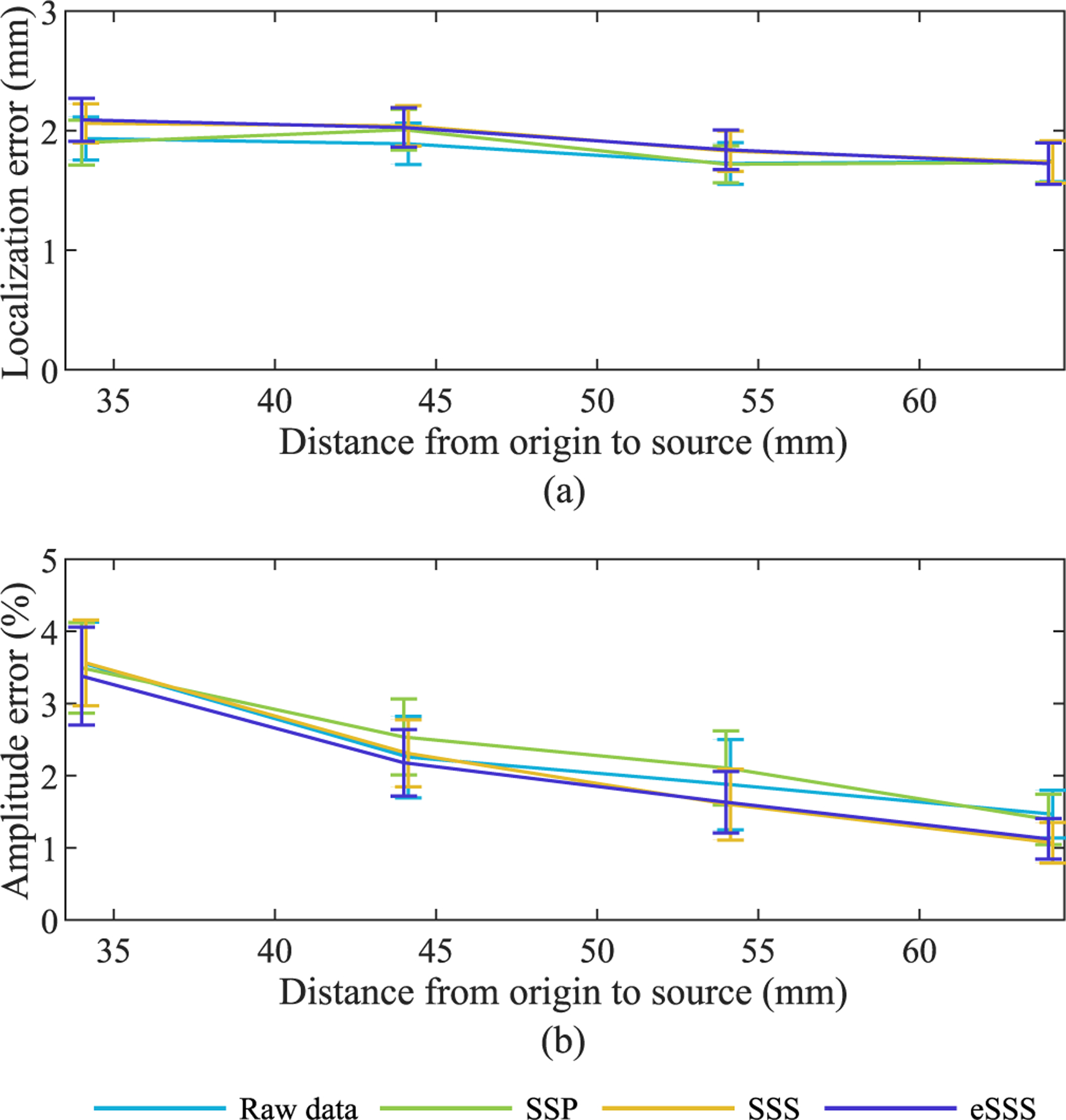
Average source estimation errors with standard error of mean. (a) Localization error. (b) Amplitude error. Measurement 1.

Source estimation errors from phantom dipole localization (Measurement 1) are shown in Fig. 5. The results indicate that at these source depths, which are consistent with real brain dimensions, eSSS introduces source estimation error comparable to that of SSS or SSP, maximum errors being only approximately 2 mm and 4%, for location and amplitude, respectively.

#### 6) Noise Sensitivity of the eSSS Model:

In order to test the appearance of the reconstruction noise in the processed data, the condition numbers of SSS vs. eSSS bases were compared (Measurement 2). While the number of basis vectors increased from 95 (SSS basis) to 103 (eSSS basis), the condition numbers remained similar (180 for SSS vs. 181 for eSSS). In line with this finding, noise levels in SSS- and eSSS-processed magnetometer data appear similar (Fig. 3 b). In the case of eSSS processing without fine-calibration information, the condition number increased slightly, from 181 to 186, resulting in a small, barely detectable increase in noise levels (data not shown).

Application of fine-calibration information and cross-talk correction in SSS modelling is crucial for obtaining good SF (see Tables III and IV). However, the embedded statistical information in the eSSS model results in decreased sensitivity to the calibration errors, as seen in a simulation (Table III, “known” interference). Table IV shows that also in the case of real data, the difference in eSSS SFs with and without fine-calibration information is only minor, given also that in both cases the eSSS processing reached approximately the sensor noise level.

#### 7) Auditory Evoked Fields:

**Fig. 6.**
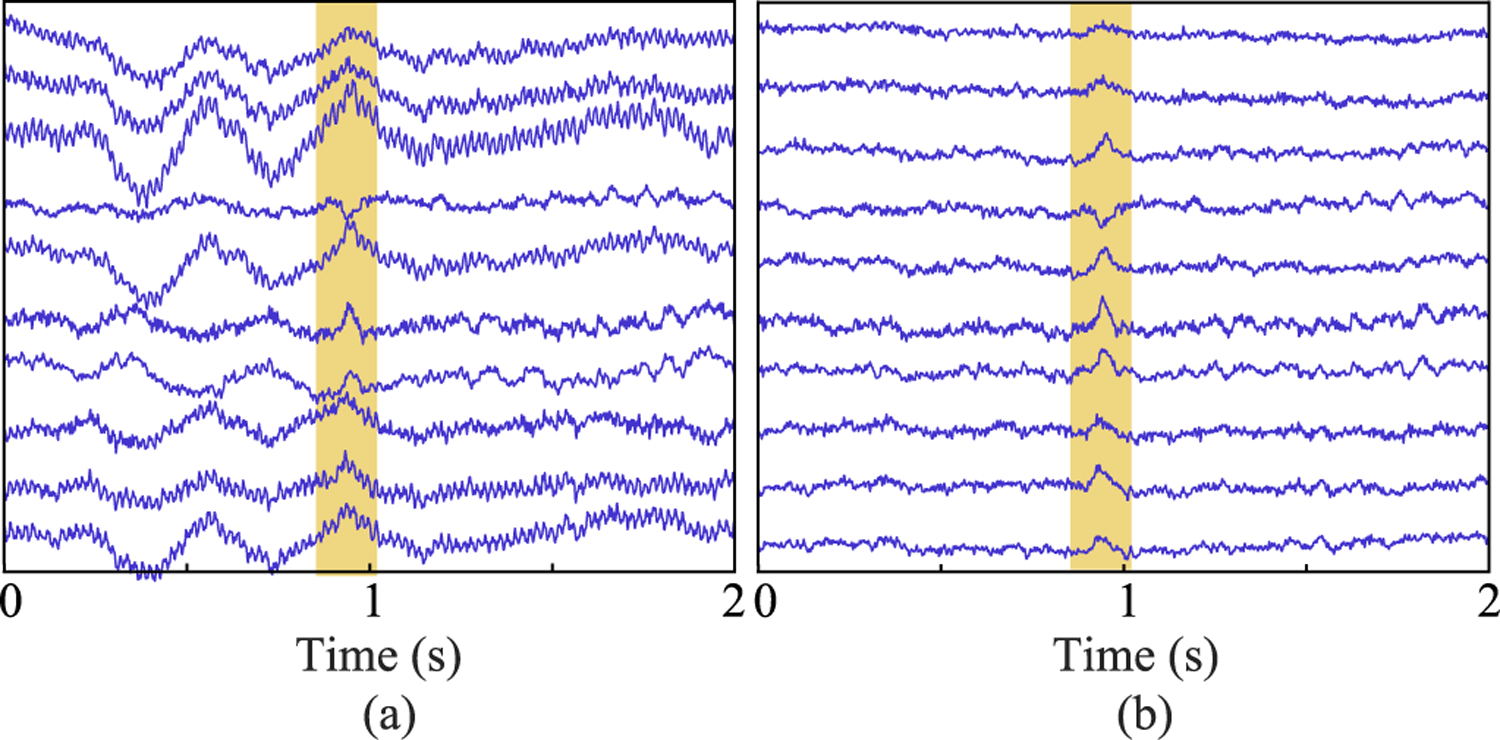
Sample of left temporal gradiometers from Measurement 6 showing single-trial auditory evoked field (highlighted). (a) SSS-processed, showing residual interference, (b) eSSS-processed. Vertical scale 400 fT/cm.

The distance between the source locations obtained from the SSS- and eSSS-processed averaged data was *<* 3 mm for AEFs from both hemispheres, and these sources localized close to the supratemporal auditory cortex, as expected, when overlaid on the subject’s anatomical MR images. Analysis of single-trial N100 responses (number of trials 49) gave the average source amplitudes of 130 nAm and 136 nAm for SSS- and eSSS-processed data, respectively. Similarly, average distances of the source locations were 9 mm and 5 mm, and maximum distances 49 mm and 15 mm, for SSS- and eSSS-processed data, respectively. In line with our simulations and phantom study, these results provide further evidence that eSSS does not introduce systematic errors to source estimation but enables more accurate source localization of single-trial data compared to SSS. This improvement of data quality is also illustrated in Fig. 6 that shows signals on sample gradiometers above the left temporal cortex after SSS and eSSS processing; while the 3-Hz interference is still visible after SSS processing, eSSS clearly suppressed this interference revealing the single auditory evoked response.

## IV. Discussion

MEG devices located in, e.g., hospital environments, and especially those in light-weight MSRs, can be exposed to strong external interference fields that should be suppressed by a very large factor to ensure reliable results regarding brain activity. The established interference suppression methods – such as SSS and SSP – do not always provide a suppression factor that is sufficient in such harsh interference conditions. Therefore, we developed a new method, eSSS, to overcome some of the limitations of SSS and SSP and to combine the benefits of the two methods.

First, we demonstrated the ability of the eSSS method to significantly improve suppression against external interference in comparison to SSS and SSP. With only “unknown” interference, eSSS reduced to SSS as shown by their equal shielding performance in our simulation. However, “unknown” interference in real data includes similar field directions as empty-MSR data due to the field-homogenizing effect of MSR and therefore eSSS performs better than SSS. With “known” data, eSSS seems to perform equally well with simulated and measured data, giving high SFs. Thus, whereas SSS suppresses both known and unknown external interference with a shielding factor of 200–300, and SSP suppresses only “known” interference but with a very high SF, our novel eSSS provides a high SF for all external interference, both known and new. Importantly, eSSS reduced the external interference to the level of sensor noise in all our test cases except when the internal active shielding was enabled.

We also demonstrated that the eSSS processing does not increase error in source estimation. Simulations, having a large range of dipole depths, showed a tendency to slightly increased source estimation error in i) deep sources (SSP processing) and ii) superficial sources (SSS and eSSS processing). The former can be understood as the spatial field patters from very deep sources start to resemble those of external fields at the level of sensors, and thus might be unintentionally projected out by SSP [4]. However, the source signal amplitude suppression visible in Figs. 1 and 3 in case of SSP, did not cause an amplitude error in source localization as the effect of the SSP projection was taken appropriately into account in source modelling. In case of phantom data, all the processing methods gave consistent results of source estimation error, showing a slight increase towards deep sources, which, however, are not as deep as in the simulations. While all the source localization errors remained below 3 mm, results from the deepest sources suggest that eSSS is better suited than SSP to process signals generated by deep brain sources, such as brain stem sources [23], in order to avoid source signal amplitude suppression.

The latter case of source localization errors on superficial dipoles, on the other hand, can be explained by the potential inadequate modelling of high spatial frequencies of the internal source signal due to truncation of the VSH expansion in (3) [6]. Superficial sources are more prone to truncation errors due to their proximity to the sensors, but *L*_in_ = 8 has been shown to be sufficient for SSS to keep error below 1 mm [6]. As eSSS extends the SSS model, the truncation error also applies to the eSSS modelling, and in our case the error remained below 2 mm. However, the results from real data at similar dipole depths do not show evidence of source amplitude bias even in most superficial dipoles. Most importantly, the 2-mm maximum error that we observed in case of eSSS processing is insignificant compared to the other error sources in MEG data, such as head digitization and localization, co-registration of MEG to MRI or source localization algorithms. Even the accuracy of the MEG phantom is likely not better than 1–2 mm. Thus, all this imply the safe use of the suggested eSSS method with all brain sources independent of their depths, and especially in cases of very deep sources.

Our results also indicated that application of fine-calibration information does not have a significant effect on the SF provided by eSSS and the residual interference after eSSS processing without fine-calibration information is negligible. This is due to the ability of eSSS to correct for the calibration inaccuracies in the directions of the interference fields used to augment the basis. Traditionally, the fine-calibration information has only been available for 306-channel MEG systems of MEGIN Oy (formerly Elekta Oy), which has limited the efficient use of SSS among other MEG systems. On the other hand, the SSS method is based on a physical model of magnetic fields that, in principle, is applicable to any multichannel MEG array and as such is not specific to any sensor configuration or sensor type. Due to its insensitivity to calibration accuracy, the eSSS method could be directly applied when the sensor geometry is not precise (e.g. manually manufactured sensors) or the fine-calibration information is not available, (e.g. data measured with any multichannel MEG system). tSSS method has been demonstrated to perform reasonably well in suppression of external interference [24] as well as deep brain stimulator-related close-by artifacts [25] also for data from MEG systems with manually-manufactured axial gradiometers, and we would assume good performance in case of external interference on this type of data with eSSS as well.

Because eSSS is a purely spatial operation on the sensor level data, we compared it to other spatial filtering methods: SSS and SSP. On the other hand, ICA which can also efficiently extract artifact or brain-related independent components (ICs) from MEG data [26], was omitted from comparisons here. This was partly due to the requirement of manual classification of the ICs, although a method for automated detection and subtraction of artifact ICs based on approximate entropy measure has been introduced [27]. Furthermore, we did not extend the comparison to spatiotemporal filtering such as tSSS, operations in the frequency domain such as spectral signal-space projection [11], nor methods in the source space such as beamformers [28], [29], due to their different working principles. This way, we wanted to guarantee the quality and generality of the comparison results and avoid dependence on, for example, tSSS parameter selection. All of the aforementioned methods, however, can be used in addition to the eSSS method to tackle different types of artifacts. Especially tSSS, being an extension of the SSS processing developed for suppression of close-by artifacts, could be applied using the eSSS basis instead of SSS (resulting in “teSSS”). Although tSSS alone can enhance the suppression of strong external interference in cases that are associated with a significant residual after SSS, the combination “teSSS” would work more efficiently as the spatiotemporal complexity of the residual signal would be reduced due to the enhanced spatial interference modeling accuracy provided by the eSSS basis. For the same reason, signal discontinuities, occasionally visible at the borders of the processing windows after tSSS processing, would disappear. Thus, data containing both strong external- and close-by interference would especially benefit from combined “teSSS”.

Unlike SSS, eSSS requires a short measurement of empty-MSR data, preferably from the same measurement session as the actual subject recording. It is generally a good practice to always perform this kind of a quality control measurement, so the task should not be considered to cause extra workload. However, unlike SSP that suppresses the interference patterns present in the empty-MSR measurement, eSSS only requires this information to correct for the inaccurate calibration information. Thus, and due to the fact that environmental interference fields tend to stay relatively constant, eSSS could also work reasonably well with general good-quality empty-MSR data obtained in a different measurement session.

Unlike in all other use cases, eSSS applied to data with IAS active resulted in small residual interference field. This is most likely due to the mismatch of spatial shapes and complexities between empty-MSR data and the data with the internal source, originating from the fact that IAS uses a subset of MEG magnetometers to define the compensation fields. When IAS is active, the compensation coils on the walls of the MSR contribute to the data by adding patterns that may not be present in empty-MSR data with IAS active or not, thus increasing the complexity of external interference patterns. To overcome this potentially reduced SF, each of the IAS compensation coils could be activated separately and the related signal vector subsequently measured. Then, these extracted signal vectors could be added to the eSSS basis as separate patterns. Even without this process, however, the residual interference would probably be low in amplitude and at least lower than that after SSS-processing. Thus, eSSS would be recommended in case of data measured with IAS active as well.

In our implementation, eSSS contains the computational SSS basis augmented by the dominating PCs from empty-MSR data, as well as frequency-specific components in case of vibration of MSR. In addition, it could be extended by any other relevant interference pattern, PCA- or, for example, ICA-based. Moreover, spatial patterns derived from subject-specific data, e.g., blinks or cardiac artifacts, could be added to the eSSS basis to provide suppression of physiological artifacts as well. Preliminary results from adding 1–2 PCs from the averaged blink-related epoch to the $S_{\text{out,e}}^{\prime }$ basis look promising. In the case of physiological artifacts, the linear independence of the eSSS basis need to be confirmed before using it in the modelling.

Whereas the SSS method is often used in MEG pre-processing, eSSS could be used to replace the basic SSS for enhanced interference suppression. In addition to the already mentioned combined “teSSS” approach, eSSS can be directly used with SSS-based head movement compensation [5], [30] or with sensor noise suppression procedures, such as cross-validation SSS [31], applied in [32], or oversampled temporal projection (OTP) [33], [34]. Different approach to the successful increase of the SSS method’s SF is the improved fine calibration by 3D-imbalance model [35], reported to increase the SF of SSS at least 100%. Although eSSS seemed to suppress purely external interferences to the level of sensor noise using traditional fine-calibration information, or even without it, application of the 3D-imbalance model in eSSS processing could further enhance the suppression in cases of extreme interference, if residual field still exists after eSSS.

As eSSS is a purely spatial operation, it could also be effectively used in online applications [36], [37] without heavy computation or need for windowing data for temporal operations such as tSSS. Therefore, in addition to enhanced data quality in off-line processing, this superior interference suppression could be easily obtained for online purposes, potentially facilitating e.g. the development of brain–machine interfaces [38], [39] even in magnetically challenging environments.

Compact optically-pumped magnetometers (OPM), sensitive enough for MEG, have recently become available, see e.g. [40]. However, most OPM sensors are read out directly, i.e., without negative feedback, which makes their calibration to depend on the magnetic field [41]. Thus, reaching calibration accuracy required for obtaining a high SF with SSS is, at least with current OPM technology, not possible. However, eSSS could potentially alleviate this problem as it could provide high SF without accurate calibration and could therefore be the method-of-choice for interference suppression in multichannel OPM devices. However, due to the reduced source-to-sensor distance in OPM technology, higher spatial frequencies of the magnetic fields can be captured, and thus higher *L*_in_ values in eSSS modelling would be required, potentially *L*_in_ *>* 10.

## V. Conclusion

The increasing interest in unaveraged measurements of brain activity emphasizes the importance of high-quality data. External interference has a substantial effect on MEG data quality as it often dominates the recorded MEG signals at frequency bands of neuroscientific interest. The eSSS method introduced here combines the advantages of the SSS and SSP methods, resulting in superior and more robust interference suppression that adapts to a changing magnetic environment and performs efficiently even when high-precision calibration of the MEG system is not available. Importantly, eSSS reduces external interference to the level of sensor noise in most cases and does not introduce bias to source localization, which we demonstrate as improved source localization accuracy of phantom measurements and unaveraged auditory evoked responses. Thus, the robust and efficient interference suppression provided by eSSS can facilitate reliable analysis of low-amplitude, single-trial brain signals even under very challenging interference conditions.

## Figures and Tables

**TABLE I:** Simulations

Simulation	Internal signal	External interference
1	-	External
2a	Brain source	”Known” external
2b	Brain source	”Unknown” external
3	Brain source	-

**TABLE II:** Measurements. Data in Measurements 1–5 Were Collected With 306-Channel TRIUX™ Systems (Megin Oy, Helsinki, Finland) Using a Sampling Rate of 1 kHz and Passband of 0.1–330 Hz. See [9] for Details on Measurement 6.

Measurement	Experiment	Shielding	Site
1	Phantom	Passive	Factory
2	External interference	Passive	Factory
3	New external interference	Passive	Factory
4	Phantom	IAS	Factory
5	Mechanical vibration	IAS	Hospital
6	Auditory evoked fields	Passive	Aalto Univ.*

* Formerly Helsinki Univ. of Technology

**TABLE III:** Shielding Factors for Simulated Data (Simulation 2)

Method	No fine calibration	Fine calibration
**Interference “unknown”**		
SSP	1.0	1.0
SSS	29	210
eSSS	30	210

**Interference “known”**		
SSP	710 000	710 000
SSS	29	210
eSSS	240 000	260 000

**TABLE IV:** Shielding Factors for Real Data (Measurement 2)

Method	No fine calibration	With fine calibration
SSP	3000	3000
SSS	42	310
eSSS	3600	4100

**TABLE V:** Shielding Factors for Real Data (Measurement 3)

Method	Interference “unknown”	Interference “known”
SSP	130	1100
SSS	220	220
eSSS	1000	1300
